# How Serious Are Health-Related Welfare Problems in Unowned Unsocialised Domestic Cats? A Study from Denmark Based on 598 Necropsies

**DOI:** 10.3390/ani12050662

**Published:** 2022-03-06

**Authors:** Ida Sofie Thuesen, Jørgen Steen Agerholm, Helena Mejer, Søren Saxmose Nielsen, Peter Sandøe

**Affiliations:** 1 Department of Veterinary and Animal Sciences, University of Copenhagen, DK-1870 Frederiksberg C, Denmark; isth@sund.ku.dk (I.S.T.); hem@sund.ku.dk (H.M.); saxmose@sund.ku.dk (S.S.N.); 2 Department of Veterinary Clinical Sciences, University of Copenhagen, DK-2630 Tåstrup, Denmark; jager@sund.ku.dk; 3 Department of Food and Resource Economics, University of Copenhagen, DK-1958 Frederiksberg C, Denmark

**Keywords:** body condition, ectoparasite, *Felis catus*, FeLV, unsocialised, FIV, free-ranging, necropsy

## Abstract

**Simple Summary:**

Domestic cats are very popular companion animals, but a significant number are ownerless. These are either socialised or unsocialised. The former can be re-homed. Unsocialised domestic cats, on the other hand, are difficult to handle and fear humans. Therefore, rehoming them is challenging. Unowned unsocialised cats are seen by many people as a nuisance, a hazard to human and animal health, and a threat to biodiversity. However, others have concerns about their welfare, although our knowledge of how well these cats actually cope is very limited. To remedy this, we studied the corpses of 598 unsocialised cats euthanised in Denmark in 2019 by two cat welfare organisations. Our findings reveal that unsocialised cats in Denmark face only a moderate load of health-related welfare problems, even in comparison with owned cats. So, although there may be other reasons to euthanise unsocialised cats, this practice may, in a place like Denmark, be difficult to justify by pointing to the welfare needs of the cats.

**Abstract:**

Free ranging unsocialised domestic cats are widely believed to suffer from a high load of welfare problems. We assessed the validity of this belief by performing necropsies on the corpses of 598 unsocialised cats, originating from all parts of Denmark, that had been euthanised by two Danish cat welfare organisations. We selected a number of variables for health-related cat welfare that could be assessed through necropsy (e.g., gross lesions, ectoparasites and body condition) or by laboratory analysis (e.g., infection with feline immunodeficiency virus (FIV), and by feline leukaemia virus (FeLV)). Each finding was classified as having either a major or minor welfare impact on the cat. More than 83% of the cats had no major finding, and 54% had no finding indicating a welfare issue at all. More than 83% of the cats had a body condition within normal range. Only 0.3% were emaciated. The most common finding was infestation with ectoparasites, with 15.9% infected with lice, 12.3% with fleas, 4.7% with ticks, and 6.7% with ear mites. FIV and FeLV were detected in 9.2% and 1.2% of the cases, respectively. The most common lesion related to the cats’ teeth. Overall, unsocialised cats in Denmark have a moderate level of health-related welfare problems.

## 1. Introduction

The domestic cat (*Felis catus*), along with the dog (*Canis lupus familiaris*), is the most popular companion animal in many Western countries [1]. However, there are also large populations of free-ranging domestic cats.

Some of these free-ranging cats are formerly owned but this group also includes so-called ‘barn cats’, i.e., cats that do not live inside the houses of their owners but live in outbuildings such as stables, barns, and garages, and may be provided with food and water. What these cats have in common is that they are typically socialised to humans and can go into shelters and be rehomed if desired.

However, some free-ranging cats are unsocialised. These animals are usually difficult to handle, fear humans and cannot be placed in a typical private home [2]—which is not to say that they cannot attach to individual humans [3]. They are sometimes referred to as ‘feral cats’, but it should be noted that cats of this sort typically are not feral in the biological sense applied to semi-wild animals living independently of humans [2]. Rather they tend to live close to humans, on whom they depend to some extent for food—some of which comes from scavenging garbage and other anthropogenic left-overs [4]. The parents of the cats may be unsocialised or socialised.

Unowned unsocialised domestic cats can be, and often are, regarded as a problem for several reasons. They are seen by many people as a nuisance, because they dig and spread their faeces in gardens and public places, and because they can be noisy and scare or harm companion cats with outdoor access [5]. They may also be looked upon as a health hazard, because they are potential transmitters of infectious agents to wild and domestic animals, and to humans [2]. Last, but not least, the unsocialised cats are regarded by some as a threat to nature and biodiversity, mainly because they are predators [6], and this perception persists even though some of the very strong claims made in this regard have been criticised [7].

On the other hand, there is widespread concern about the welfare of unsocialised cats, and a growing number of animal welfare NGOs are working to improve their welfare through various initiatives. For example, efforts are made to promote strategies for the control of unowned unsocialised cat populations involving no, or limited, killing of the cats, notably through support for programmes of ‘trap, neuter and return’ (TNR) [2]. These initiatives are not extensive, however, and in many countries the cats are more likely simply to be euthanised, because this is relatively cost-effective—at least, in the short term—and because there is a perception that the cats may be better off dead than being left to fend for themselves.

Evidence-based knowledge and understanding of how unsocialised cats fare is currently limited. In a focused literature search we identified 15 studies that directly or indirectly focus on the welfare of unsocialised cats, from Australia [4], Belgium [8,9], Canada [10], Israel [11], Iran [12], Italy [13], France [14], Japan [15,16], Malaysia [17], Mexico [18], Puerto Rico [19], and the USA [10,20,21]. These differed in their methodologies: three used visual observation of the cats (which was sometimes supplemented with photo or video capture) [15,16,19], one was based on questionnaires completed by feeding hosts [11], six examined the animals and/or took blood samples from them during anaesthesia, mostly as part of a TNR programme [8,10,13,14,20,21], and four investigated only, or mainly, euthanised cats [4,12,17,18]. One, based on TNR [9], also included necropsy of euthanised cats.

Nearly all the studies lacked a clear focus on unsocialised cats and their welfare. Some explicitly included a range of different kinds of cats, including owned companion animals. Even in the studies with a focus on free-ranging cats, clear distinctions were rarely drawn between socialised and unsocialised unowned cats. Also, some of the studies looked at managed unsocialised cats from ‘cat colonies’. 

In addition, many of the studies were not focused on cat welfare as such. They investigated the unowned cats as potential vectors of viral disease [8,9,10,14,21] or parasites [12,17,18,20]. One study [13] focused narrowly on *otitis externa* and used unowned cats mainly to study the disease and assess the risks to companion cats with outdoor access.

The remaining five studies, which did have a clear focus on the welfare of unowned cats [4,11,15,16,19], presented very different conclusions to which we will return in the discussion. All five had two main limitations: (1) nearly all the cats were from urban areas, and (2) the extent to which the cats were socialised or unsocialised was unclear.

We have attempted to avoid these limitations of previous studies in the current investigation. First, we obtained a large number of unsocialised cat corpses. Secondly, we focused throughout on their welfare. Since the cats were already euthanised when we received them, we could not study all parameters of relevance to welfare assessment. Notably, we were unable to assess behavioural evidence of positive or negative welfare, and also unable to look at environmental factors that may give rise to various kinds of discomfort in the cats. However, we studied two of the four domains, health and nutrition, which, according the influential Five Domains Model of animal welfare [22], can affect the mental state of an animal. For brevity, we here combine these under the label ‘health related welfare’.

Thus, we undertook a study of unsocialised domestic cats in Denmark based on necropsy and subsequent analysis of parameters of relevance to the assessment of an animal’s health-related welfare.

## 2. Materials and Methods

The cats were supplied by two Danish animal welfare non-governmental organisations (NGOs): Dansk Dyreværn Århus, which covered the area in and around the city of Aarhus, and Kattens Værn, which has agreements with 45/98 of the Danish municipalities on the capture of unsocialised cats. 

Danish national legislation permits cats with no owner or whose owners cannot be found, to be caught and euthanised provided this is done under the supervision of a veterinarian and is permitted by the local municipality where it takes place. Trained staff working for NGOs (so-called “cat inspectors”) are authorised to trap such cats when citizens in the municipalities make complaints.

In 2019, the total number of cats captured by cat inspectors from Kattens Værn was 5775. Of these, 7.9% went into a TNR programme, where local feeding hosts took responsibility for the cats. Note that only a small number of the cats caught could go into a TNR programme due to very restrictive Danish regulations for releasing of cats. Lactating cats without their kittens, accounting for 1.4%, were released, and 20% were socialised and ended up either in a shelter for adoption or were returned to their owners. However, 64.9% were based on their behaviour classified as unsocialised, not eligible for TNR, and were euthanised. Where there was a degree of uncertainty, the cats were taken to a shelter for observation for 1–2 weeks. Only cats showing clear behavioural signs of being unsocialised were euthanised on the spot. Thus, in total 3747 unsocialised cats were euthanised in 2019 (personal communication, Lone Nielsen, former CEO of Kattens Værn).

During the study period (1 February to 15 September 2019) approximately 800 of these 3747 cats were collected with the aim of ensuring geographical representation across Denmark (except the islands not connected to the mainland by bridge). Initially, each cat was anesthetised with an intramuscular injection of a mixture of tiletamin hydrochloride and zolazepam hydrochloride (Zoletil Vet, Virbac, Kolding, Denmark). To facilitate this the foam rubber-covered roof of the trap was pressed against the cat to immobilise it. The cat was then left in the trap and watched until fully anesthetised. Following this it was removed from the trap and euthanised by intracardial injection of an overdose of pentobarbital. Death was confirmed by auscultation of the heart. The procedure was performed by veterinary-trained cat inspectors. Once death had occurred, the cats were placed in black plastic bags, which were subsequently labelled with zip codes and stored in freezers (–20 to –30 °C) by the cat inspectors. The cats were categorised as belonging to one of the following five NUTS-2 regions: The North Denmark Region, The Central Denmark Region, The Region of Southern Denmark, The Region Zealand, and The Capital Region of Denmark [23]. We aimed to ensure an even distribution among the regions, but otherwise the collection was random. For capacity reasons, only 598 of the cats were included in the study. Because all were placed in black plastic bags before sampling, the state of each cat was considered blinded to the person selecting the cats for necropsy (I.S.T.). Each cat was assigned an identification number.

It should be stressed that the cats involved in the present study would have been euthanised whether we had conducted our study or not, and that the project therefore had no direct or indirect impact on decisions about cat euthanasia. 

For human health and safety reasons, all the cats were frozen for at least 72 h at −80 °C to deactivate any eggs of the zoonotic parasite, *Echinoccus multilocularis*. The carcasses were then examined after thawing. The post-mortem examination proceeded in four steps: (1) sampling of ectoparasites, (2) age estimation, (3) necropsy, and (4) follow-up laboratory analyses. The same observer (I.S.T.) scored all cats. 

### 2.1. Examination for Ectoparasites

To collect any lice and fleas the carcass was held over a white A4 sheet of paper, and the fur was scratched, using fingers, 8 times down the back, 8 times on each side, 8 times on the hind part and 8 times on the neck. The plastic bag in which the cat had been kept during storage/freezing was also emptied over the paper. Parasites and debris were collected from the paper with a 4–5 cm × 1.8 cm piece of transparent tape, which was placed on a microscope slide (76 × 26 mm). The surface of each cat was also examined visually for ticks that were removed manually for identification. 

To detect ear mites, cerumen was collected from both ears using a separate cotton swab for each ear. The swabs were incubated in 10% KOH for 24 h to dissolve the cerumen before the sample was examined under the dissection microscope. In samples with large amounts of cerumen, the cerumen was mechanically manipulated to accelerate the dissolving process. 

Ectoparasites were examined and identified [24,25,26,27] using a dissection microscope at 16–80× magnification. Cats were registered as positive for a particular parasite if at least one specimen was detected; otherwise they were registered as negative.

The recovered ectoparasites were counted for all cats, except four that were so heavily contaminated with a mixture of blood, urine and thawing fluid, spread in the fur, that checking for fleas and lice was not practicable. These cats were still examined for ticks, however. Time limitations also meant that only 60% of the cats were examined for ear mites.

### 2.2. Age Estimation

Age was recorded as either juvenile or adult based on the presence (juvenile) or absence (adult) of juvenile teeth.

### 2.3. Necropsy

All necropsies were performed by I.S.T., sometimes aided by veterinary students under supervision of I.S.T. The carcass was placed on its back and an incision was made through the skin in the ventral midline from the mandibular angle to the penis (males) or vulva (females). The skin on the medial side of the limbs was transected with the incisions proceeding from the midline to the middle of the metacarpus/metatarsus. The skin was then detached from the underlying structures, so that it was only attached along the back of the carcass. A section was then made in the ventral midline of the abdominal wall to allow the internal organs to be inspected, and the thorax was opened by clipping the ribs with a scissor.

The sex was recorded as intact female, intact male, neutered female or neutered male, and females were classified as pregnant or non-pregnant following inspection of the uterus.

The amount of subcutaneous, thoracic and abdominal fat was assessed, and the body condition categorised as emaciated, thin, normal, moderately overweight, or obese. Emaciated cats completely lacked bodyfat and had serous fat atrophy of, for example, the coronary fat. Thin cats had mainly abdominal fat with only a sparse amount of subcutaneous and thoracic fat. Cats with moderate overweight had abdominal, thoracic and subcutaneous fat. Obese cats had abundance abdominal, thoracic and subcutaneous fat. 

A necropsy was performed, except that the central nervous system was not inspected. The necropsy included inspection and palpation of all organs and the opening of the ventricle, lungs, heart, kidneys, spleen, liver, pancreas, uterus, bladder and joints in accordance with Jensen et al. [28]. Histopathology was not undertaken due to complications arising from freeze-thaw artefacts.

### 2.4. FIV/FeLV Testing

Before the completed thawing of a carcass, a 2–3 cm piece of the femur (diaphysis) was sampled using an oscillating saw (Aesculap, Inc., Center Valley, PA, USA). Between each cat the blade was cleaned and then disinfected for 2 min with Virkon S (Antec International Limited, Sudbury, UK) and 2 min with 70% ethanol. The femoral specimen was transferred to a 25 mL tube and stored at −80 °C until being shipped to a laboratory (LABOKLIN, Bad Kissingen, Germany) for testing. The testing used real time polymerase chain reaction methods modified from Klein et al. [29] for FIV and Langhammer et al. [30] for FeLV. Cats were then recorded as either positive or negative for each virus.

### 2.5. Classification of Findings Regarding Their Expected Impact on Cat Welfare

To estimate the impact of each finding on the cat’s overall welfare, abnormal findings were divided into those with what was expected to be a limited, or ‘minor’, impact and those with what was expected to be a more severe, or ‘major’, impact on the cat. Normal findings were categorised as ‘none’. As the present study did not include clinical examination or observation of the cats before euthanasia, impacts were determined only by post-mortem findings and were, therefore, to a certain extent hypothetical. The criteria against which findings were grouped into the welfare impacts none, minor and major are shown in Table 1.

Cats with normal amounts of body fat were classified as having no weight related welfare problem. Cats with moderate overweight were included in the group with no weight related welfare problems because while moderate overweight may be seen as a risk for problems linked to obesity in cats living with human owners [31], in free-ranging unsocialised cats it can serve as a buffer preventing the cats from entering a catabolic state [32]. On the other hand, being thin but not emaciated was classified as a minor problem for the cats, because unsocialised cats in this body condition are less well equipped than heavier cats to deal with food shortage. That emaciated or obese cats have significant welfare impairment is well documented in the literature [33].

Lesions were scored on a case-by-case basis. The rationale for each scoring is given in the Section 3.

Testing positive for either FIV or FeLV was considered a minor welfare impact if no corresponding lesions were observed. Cats can be latent carriers of FIV and FeLV without clinical signs and no known impact on welfare [34,35]. However, the welfare impacts on the cats were grouped as major, if lesions were present, i.e., if the cat was diseased. 

Cats were grouped into minor and major for all ectoparasites based on numbers of parasites observed. The severity of flea and tick infestations was defined in line with previous recommendations [36]. Thus, the cat flea feeds on blood and is a common cause of hypersensitivity dermatitis and scratching in cats [37]. Individual fleas may not persist long due to cats’ natural grooming behaviour [38], but fleas ingested through grooming do potentially transmit a range of pathogens [39]. This may also happen during the ticks’ blood meal [40], but we do not know how widespread the pathogens are and how frequent a cause of disease they are in Denmark. Guidelines have not been defined for lice and ear mites, and severity was therefore arbitrarily classified here based on the range of the infestation levels. Lice may cause dermatitis, itching and alopecia, and they can be highly irritating to the host as a result of their movement through the fur [41]. Ear mites may cause *otitis externa* [13], but the severity of clinical signs may not be clearly linked to infestation levels [42]. This may in part reflect the fact that complete recovery of all mites from the ears is difficult. We therefore decided to apply, for both parasites, a conservative severity scale equal to that for the ticks.

### 2.6. Statistical Analyses

Prevalence for each finding was calculated, and the resulting number of major and total findings per cat were then summarised. For example, a cat with a lice count >10 and no teeth would have had two major findings, while a cat with FIV and a lice count >10 would have had one major and two total findings.

As mentioned above, there were missing values for some conditions. Imputation was performed, using the mice-package in R [43], by sampling from existing values using the distribution in data. The imputed data were used to summarise the number of minor and major findings per cat, and not to provide estimates on individual cats. Prevalence estimates were provided with 95% confidence intervals, using the binconf()-function in R with the Wilson method [44].

## 3. Results

### 3.1. Study Population

Demographic data on the necropsied cats are shown in Table 2. The majority (81%) were categorised as adult, with an almost equal prevalence of males and females. Just 11% of the cats had been neutered. Among the intact females, 31% were recorded as pregnant (Table 2). 

### 3.2. Post-Mortem Findings

Determination of body condition showed that only two cats (0.3%) were emaciated. Most of the animals (83.1%) had normal body condition, while 11% were scored as ‘thin’ and 5.5% as moderately overweight (Table 3).

Examination for FIV and FeLV showed that these infections were not very common, with 9.3% and 1.2% positive cases, respectively (Table 3). 

Gross lesions were present in 172 cases (29%). Lesions were most common in the teeth (17%) in the form of lost or broken teeth. Bone fractures, which were uncommon, included multiple acute rib fractures *(n* = 1), absence of distal parts of the left/right antebrachium (*n* = 1) and loss of part of the tail (*n* = 3). Lesions that had caused amputation were associated with the development of an inflamed granulation tissue at the amputation site. Lesions in the internal organs (*n* = 82) consisted of a variety of pathological conditions, including hepatic steatosis, chronic renal infarcts and other focal or localised fibrotic lesions, unilateral renal hypoplasia or dysplasia or hydronephrosis with contralateral renal hyperplasia, urolithiasis (no cases with obstruction), polycystic kidneys, diaphragmatic intestinal hernia and a case of foreign body in the ventricle (Table 3).

The fur of four cats was significantly contaminated with a mixture of blood, urine and thawing fluid. These cats were submitted as pairs in two plastic bags. The necropsy did not reveal a specific cause of bleeding but indicated that the blood had oozed from the nose of one cat in each pair. Bleeding from the nose sometimes accompanies euthanasia (personal information, cat inspector Jan Flesborg). As no lesions were present that could explain the presence of blood, we concluded that oozing of blood from nose was a post mortem event. 

The most common ectoparasites were cat-chewing lice (*Felicola subrostratus*) and fleas at 15.9% and 12.3%, respectively. The fleas were made up of 71% cat fleas (*Ctenocephalides felis*) and 29% fleas belonging to the subfamily Ceratophyllinae (e.g., bird fleas). Ear mites (*Otodectes cynotis*) were only detected on 6.7% of the cats, and 3.9% of the cats had ticks (*Ixodes ricinus*). Median infestation intensities were 3 lice (max. 79), 1 flea (max. 12), 2 ear mites (max. 17) and 1 tick (max. 4) per cat (Table 3). Tick stages included 9 larvae, 2 nymphs and 17 adults.

### 3.3. Welfare Classifications of Post-Mortem Findings

In accordance with our welfare classification, 0.3% of cats were classified as having a major problem relating to their body condition because they were emaciated, and 11% were classified as having a minor problem because they were thin. 

None of the cats that tested positive for FIV or FeLV had developed lesions consistent with these infections, and these infections were therefore considered to have had minor welfare impact on the cat.

The lesions that were detected were scored case-by-case. Unilateral hydronephrosis was recorded as major, since hydronephrosis may be painful [45]. Urolithiasis was treated as major in males, but minor in females due to the risk of males developing urethral obstruction. This difference of classification was applied even in cases where no obstruction was actually present. Broken or lost molars, premolars or canines were taken to have a major impact on welfare, while loss of incisors was considered minor. Chronic and focal/localised lesions were generally classified as minor, and acute, chronic active and widespread lesions as major. Diaphragmatic hernia was recorded as a major welfare issue because the displacement of intestines into the thorax compromises respiration. By contrast, non-incarcerated abdominal hernia was recorded as minor. Diarrhoea was recorded as major because its sequelae (e.g., dehydration and acidosis) may lead to severe problems and even, in the case of kittens, be fatal [46]. All persistent or acute bone fractures were recorded as major to reflect the pain with which they are normally associated [47], while healed chronic lesions were considered minor. Most lesions of the internal organs were considered to have a minor impact on welfare, but lesions like hydronephrosis, urolithiasis (in males), and polycystic kidneys, diaphragmatic intestinal hernia and a case of foreign body in the stomach, were categorised as having a major impact.

As regards parasite infestations, only lice and ear mites were found at levels considered to have a major impact on welfare. Severe infestations ranged from 11–79 lice and 11–17 ear mites.

Of the 598 cats we examined, 54% (95% CI 50; 58%) were found to be without conditions having an adverse effect on their welfare, and 83% (95% CI 81; 87%) were either normal or had only conditions with a minor impact on welfare. Thus, 17% of the animals examined had a condition that was considered to have had a major impact on their welfare. Assessment of the individual conditions with a major impact on welfare showed that only 2% (14) of the cats had had two such conditions. None of the cats had had more than two conditions with major impact (Table 4).

## 4. Discussion

### 4.1. Contribution of Study

To our knowledge this is the most comprehensive study of health-related welfare in unowned unsocialised cats to date. It is unique in only including unsocialised cats which were classified as such by trained observers by means of the cats´ characteristic behaviour when being handled. All the other studies of unowned cats we were able to find [4,11,15,16,19] examined both unsocialised and socialised cats. Finally, our study is distinctive in its coverage of a cat population spread across a broad range of geographical locations.

### 4.2. Discussion of Main Findings Compared to Other Studies

Many extant studies of unowned cats restrict their focus either to viral disease, notably FIV and FeLV, or to ectoparasitic infections. They probably do so because these conditions are viewed as potential problems for privately owned cats. Where there is an overlap, it is of interest to compare the findings of these studies with what we have found.

Regarding viral infections, the literature on unowned cats contains a rather wide range of findings on the prevalence of FIV and FeLV. However, our findings of 9.3% FIV and 1.2% FeLV sit broadly within the range reported in other studies: 10% FIV, 5% FeLV [8]; 18.8% FIV, 0.7% FeLV [9]; 3.6% FIV, 3.6% FeLV [10]; 15.99% FIV, 0.93% FeLV [14]; 5.2% FIV, 3.3% FeLV [21].

Regarding ectoparasitic infestation, our findings are generally within the range presented in previous studies of unowned cats. Our findings on the incidence of ticks, ear mites and especially fleas were generally at the lower end of the scale on which the findings of previous studies range, while louse infestation was higher than previously recorded [17,18,20,48,49]. With respect to relative levels of infestation, the range for ticks was as expected, but it was low for fleas as compared with that in owned cats [50,51,52]. Similarly, our ear mite findings appeared to be low, though quantification is difficult and only a few studies are available for comparison [42,49,53]. In contrast, on a few cats lice were found in what appeared to be high numbers, but this may reflect that previous counts related to owned cats [50,51,52]. The entire life cycle of the louse can be completed on the host [27], and infestations can therefore potentially reach high levels (at least, in the short-term), as was the case with a few of the cats in our study. Flea eggs are typically deposited in the cat’s environment (e.g., in areas where the cat rests), and as the environment may vary a great deal for unsocialised cats [54] flea transmission may not be a high risk. Many fleas are opportunistic rather than strictly host-specific, and it is common to find a range of flea species on cats [18,20,55].

As we mentioned in the Introduction, we were able to find five studies that had a clear and specific focus on the welfare of unowned cats. 

Two Japanese studies [15,16] based on observations of cats living in densely populated city areas over two or three year periods concluded that about 50% and 28%, respectively, of the cats were in a state of poor health. The second of the studies included a comparison with managed unowned cats in a TNR programme and concluded that the latter were healthier.

One study from Israel [11] based on the survey responses of people who fed cats in an urban area reported that 70% of the cats were neutered, and that ‘1.6% (54/3337) of the cats were limping, 2% (67/3337) suffered from systemic disease, 4% (135/3337) had skin lesions, and 3.9% (130/3337) were cats suffering from chronic disability’. 

An Australian investigation [4] looked at a sample of 188 cats that were euthanised because they were unsuitable for rehoming—either because they had incompatible temperament (i.e., exhibited behavioural problems caused very probably by their not being socialised) or because they had untreatable medical conditions. Of these cats, 78% came from urban, suburban or commercial sites, while 22% came from refuse tips in rural areas. It was found that 7% of the animals had notable health issues. ‘These included life-threatening injuries in seven cats (four stray cats were struck by motor vehicles, one was euthanised because of severe facial burns, one cat had a badly damaged forelimb, and another had a 100-mm-length axilla abscess probably due to fighting), GI blockages were detected in six cats (four cats with abnormally distended, ischaemic GI tracts, two cats with extremely large furballs of 92 and 171 g), and one cat had a liver tumour which threatened long-term survival.’ The cats all had good to excellent body and fur conditions with over 80% of them scoring 3 on a scale from 1 to 3, and none scoring 1. All the cats were infested with fleas, but this did not seem to affect their fur condition to a major extent.

Lastly, a study from an urban area in Puerto Rico [19] made use of a two-day visual encounter survey to observe 178 cats, 70% of which were neutered. It was found that 21% of the cats ‘presented visible health issues, including scabies, scars, hairless areas, black spots in the mouth and/or around the eyes and ears, and blindness, while some cats were extremely underweight, despite the fact that large amounts of food were provided’. No information is provided in the study on the distribution of these conditions.

Our results are in line with those presented in the studies from Israel and Australia. However, they contrast with the findings in the two studies from Japan in that we found that a much smaller relative number of the cats we examined had major health issues. Like the Australian study, we found that very few cats had a body condition giving rise to concerns. The study from Puerto Rico was less specific as regards its findings on health-related welfare issues, but since only 21% of the cats ‘presented visible health issues’ its findings were not far removed from ours (an exception is the Puerto Rican finding that ‘some cats were extremely underweight’).

Our study provides good evidence that, although some unsocialised cats in the areas we studied suffer from health-related welfare problems, the majority enjoy a reasonably good level of health-related welfare. Whether our findings can be generalised to unsocialised cats living in other areas, with climatic conditions that differ from those in temperate Denmark, and with different regimes for managing these cats, is an open question.

Of course, some may argue that this group of cats still has a higher level of health-related welfare impairment than that seen in cats kept as companions. However, while companion cats are likely to fare better when it comes to some of the problems studied here, such as viral infections and parasite load, they also face several significant welfare problems of their own. So far as weight status is concerned, for example, they seem to be worse off than unowned unsocialised cats. A European study [56] has found a level of obesity of 8% among companion cats, and data from the US have indicated that around a third of the companion cats are obese [57]. A non-negligible share of companion cats also seems to have significant pathological conditions. Thus, a large Finnish study [58] found that 28% of companion cats suffered from dental and oral disease, and that 12% had a disorder of the urinary system. 

### 4.3. Limitations

Our study of the welfare of unsocialised domestic cats examined their health and their nutrition, which are two of the five domains of animal welfare [22]. We did not examine the behaviour and environment of the cats, and therefore we were only able to consider the mental domain partly through the impacts upon it of health and nutrition. Since the cats we studied were, as far as we could tell, able to roam freely and capable of choosing to interact as they wished, their behavioural needs may to some extent have been fulfilled. However, there may still have been behavioural issues giving rise to problems with fear and distress, and there may have been deficiencies in the environment creating various forms of discomfort. Some of these potential welfare problems could have been tracked by measuring physiological stress reactions in the cats. However, even if it had been practically feasible to do this, it would not have been relevant to measure acute stress in the trapped cats, since the stress reaction measured would reflect reactions to being trapped. In theory, it might have been possible to measure markers of long-term stress in the faeces of the cats, but this probably would not have been feasible, because the cats were not frozen immediately after their death. 

We chose to focus on a small number of pathogens, namely FIV and FeLV, and four ectoparasites. Many others could have been looked at, including endoparasites. Endoparasites were indeed assessed for a small part of the sample, and some may have had a major impact on the cats. An example here is *Aelurostrongylus abstrusus*, which can cause respiratory challenges. However, little evidence of *A. abstrusus* was observed in the lungs in the necropsies reported here, as would otherwise have been expected [59].

The prevalence of endoparasites will be reported in a separate study on a subsample of the cats. To report it here we would have needed either to conduct an extensive imputation covering about 2/3 of the data to provide the summary scores, or to exclude 2/3 of the data. Neither option was attractive. 

The cats were selected on the basis of requests to the Danish cat NGOs Kattens Værn and Dansk Dyreværn Aarhus, both of which help to regulate the population of unsocialised cats in Denmark. The requests for regulation came from individual citizens, housing or other corporations, or from local authorities, who for one reason or another had been dissatisfied about unsocialised cats roaming in their neighbourhoods. This meant that a broad sample of unsocialised cats are represented in the study based on a broad spectrum of reports. Given that an effort was made to cover a wide geographical spread, the sample is as representative of Denmark as is practically possible. However, sampling bias cannot be excluded. One of the more important sources of bias to be considered here is selection bias in the reporting of the cats. This could have gone in two directions: healthy cats may have been reported more often because they were more active; on the other hand, unhealthy cats may have been reported more often because the people making the reports felt pity for them. We also recognise that very sick or dying cats will not have been caught. They would have been hiding. We have no information as to which bias is the more likely to have occurred.

Finally, we have framed our summary of the welfare problems around a distinction between minor and major welfare impacts. This distinction was made a priori as regards body condition, viral infections and ectoparasite levels, and on a case-by-case basis for the patho-anatomical findings. Of course, a degree of subjectivity will be involved in such a classification, and the category ‘major’ is very broad. It is our view, however, that without this distinction the results would be more misleading than they are now. For those who disagree with our scoring, we present the underlying numbers.

## 5. Conclusions

The study shows that unowned unsocialised cats in Denmark do not face a very high load of health-related welfare problems. More than 83% of the studied cats had no major findings and over 55% had no findings at all. Of the 17% cats with major findings, only a small number had two findings with major impact, and no cats had more than two such findings. More than 83% of the cats had a normal body condition. Just 11% were underweight, only 0.3% were emaciated. None were obese. Some of the most common findings were mild infestations with ectoparasites. FIV and FeLV infections were rare, at 9.3% and 1.2%, respectively. Among other patho-anatomical conditions, dental disorders (16.4%) were found to be the most common. Even when it is compared to that found in owned cats, the load of welfare problems here is not very large. There may be a number of self-interested, anthropocentric reasons to routinely euthanise unsocialised cats, but this practice is difficult to justify by pointing to concerns about the welfare of the cats themselves.

## Figures and Tables

**Table 1:** Classification of findings in terms of expected impact on animal welfare.

Type of Finding		Expected Impact on the Cat’s Welfare		
		None	Minor	Major
Body condition		Normal, moderate overweight	Thin	Emaciated, obese
Viral infections	FIV	Negative	Positive, no FIV lesions	Positive, FIV lesions
	FeLV	Negative	Positive, no FeLV lesions	Positive, FeLV lesions
Gross lesions		None	Scored case-by-case	
Ectoparasitic load	Lice	0	1–10	≥11
	Fleas	0	1–20	≥21
	Ticks	0	1–10	≥11
	Ear mites	0	1–10	≥11

FIV: feline immunodeficiency virus; FeLV: feline leukaemia virus.

**Table 2:** Demography and other population data of the 598 necropsied unsocialised cats.

Factor	Level	Frequency (%)
Geography	Capital Region of Denmark	38 (6.4%)
	Central Denmark Region	127 (21.2%)
	Region of Northern Denmark	98 (16.4%)
	Region of Southern Denmark	162 (27.1%)
	Region Zealand	171 (28.6%)
	Unknown	2 (0.3%)
Age	Adults	485 (81.1%)
	Juveniles ^#^	113 (18.9%)
Gestation *	Pregnant	81 (31.4%)
	Not pregnant	177 (68.6%)
Sex and neutralisation status	Female—intact	258 (43.1%)
	Male—intact	276 (46.2%)
	Female—neutered	39 (6.5%)
	Male—neutered	25 (4.2%)

^#^ including 11 suckling kittens that were euthanised together with their dams; * data only for intact females (population at risk).

**Table 3:** Prevalence with associated 95% confidence interval (95% CI) for each welfare condition for 598 unsocialised cats in Denmark in 2019.

Condition	Level	Number and Frequency (%)	95% CI
Body condition	Obese	0 (0%)	0.0; 0.6
	Moderate overweight	33 (5.5%)	4.0; 7.6
	Normal	497 (83.1%)	79.9; 85.9
	Thin	66 (11.0%)	8.8; 13.8
	Emaciated	2 (0.3%)	0.1; 1.2
Feline immune-deficiency virus *	Negative	536 (90.7%)	88.1; 92.8
	Positive	55 (9.3%)	7.2; 11.9
	Not tested	7 (1.2%)	
Feline leukaemia virus *	Negative	584 (98.8%)	97.6; 99.4
	Positive	7 (1.2%)	0.6; 2.4
	Not tested	7 (1.2%)	
Urogenital system	Normal	553 (92.5%)	90.1; 94.3
	Renal infarcts, unilateral hypo- or dysplasia, focal cortical fibrosis, urolithiasis in females, heterogenous urine	36 (6.0%)	4.4; 8.2
	Urolithiasis in males, polycystic kidneys, hydronephrosis	9 (1.5%)	0.8; 2.8
Oral cavity	Normal	499 (83.4%)	80.3; 86.2
	Loss of incisors,	33 (5.5%)	4.0; 7.6
	loss of molars, premolars or canines Damage to any tooth	66 (11.0%)	8.8; 13.8
Lower gastro-intestinal tract	Normal	585 (97.8%)	96.3; 98.7
	Enlarged mesenteric lymph nodes, abdominal hernia	6 (1.0%)	0.5; 2.2
	Diarrhoea, obstructive foreign bodies, diaphragmatic hernia	7 (0.6%)	0.4; 2.4
Respiratory system	Normal	595 (99.5%)	98.5; 99.8
	Enlarged lymph nodes	3 (0.5%)	0.2; 1.5
	Other findings	0 (0.0%)	0.0; 0.6
Skin	Normal	574 (96.0%)	94.1; 97.3
	Ulcerations (often bites), alopecia, othaematoma	22 (3.7%)	2.4; 5.5
	Larger ulcerations	2 (0.3%)	0.1; 1.2
Liver	Normal	577 (96.5%)	94.7; 97.7
	Steatosis, focal fibrosis	21 (3.5%)	2.3; 5.3
	Other findings	0 (0.0%)	0.0; 0.6
Skeleton	Normal	587 (98.2%)	96.7; 99.0
	Healed fractures	3 (0.5%)	0.2; 1.5
	Loss of tail, loss of antebrachium, other fractures	8 (1.3%)	0.7; 2.6
Lice	0	499 (84.1%)	81.0; 86.9
	1–10	77 (13.0%)	10.5; 15.9
	>10	17 (2.9%)	1.8; 4.5
	Not tested	5 (0.8%)	
Fleas	0	520 (87.7%)	84.8; 90.1
	1–20	73 (12.3%)	9.9; 15.2
	Not tested	5 (0.8%)	
Ticks	0	570 (96.1%)	94.2; 97.4
	1–10	23 (3.9%)	2.6; 5.8
	Not tested	5 (0.8%)	
Ear mites	0	335 (93.3%)	52.0; 59.9
	1–10	22 (6.1%)	2.4; 5.5
	>10	2 (0.6%)	0.2; 2.0
	Not tested	239 (40.0%)	

* Only 591 cats were tested, as 7 samples were lost in a freezer breakdown.

**Table 4:** Distribution of total findings and major findings per cat among 598 unowned, unsocialized cats in Denmark in 2019. The findings were summarised based on pathoanatomical findings, findings of ectoparasites and FIV and FeLV. Data were imputed for some cats, because FIV and FeLV *(n* = 7), ectoparasites *(n* = 5) and ear mites *(n* = 239) were missing.

Type of Findings	Number of Findings Per Cat	Prevalence	95% Confidence Interval
Minor and Major	0	54%	50–58%
	1	35%	32–39%
	2	7.7%	5.8–10.1%
	3	2.0%	1.2–3.5%
	4	0.3%	0.09–1.2%
Major	0	83%	81–87%
	1	15%	12–18%
	2	2.3%	1.4–3.9%
	3	0.0%	0.00–0.64%
	4	0.0%	0.00–0.64%

## Data Availability

All raw data from the necropsies are available in the Supplementary Materials S1-file, Table S1.

## Supplementary Materials

The following is available online at https://www.mdpi.com/article/10.3390/ani12050662/s1: Table S1: Health findings from 598 unowned unsocialised cats in Denmark, including the raw data file used for the prevalence estimations.
